# Smoking, nicotine and pregnancy 3 (SNAP3) trial: protocol for a randomised controlled trial of enhanced support and nicotine replacement therapy (NRT) offered for preloading, lapse recovery and smoking reduction in pregnancy

**DOI:** 10.1136/bmjopen-2025-109568

**Published:** 2025-11-13

**Authors:** Katarzyna A Campbell, Miranda M Clark, Alan A Montgomery, Christopher Partlett, Anne Dickinson, Lucy Bradshaw, Matthew Jones, Yue Huang, Paul Aveyard, Yimin Jiang, Christopher M Holmes, Tim Coleman

**Affiliations:** 1 Unit of Lifespan and Population Health, University of Nottingham School of Medicine, Nottingham, UK; 2 Nottingham Clinical Trials Unit, University of Nottingham School of Medicine, Nottingham, UK; 3 Department of Primary Care Health Sciences, University of Oxford, Oxford, UK

**Keywords:** Tobacco Use, Pregnancy, Smoking Reduction, Randomized Controlled Trial

## Abstract

**Introduction:**

Nicotine replacement therapy (NRT) helps pregnant women quit smoking. Usual National Health Service (NHS) cessation care in pregnancy starts only after women stop smoking and comprises behavioural support and NRT. NRT is stopped if women restart smoking. We hypothesised that NRT would have a bigger effect on cessation in pregnancy if used: (1) to reduce smoking before quitting (‘preloading’), (2) during brief smoking lapses after quitting and (3) to help those who cannot stop smoking, to reduce instead.

**Methods and analysis:**

A two-arm parallel group, open-label, multicentre, assessor-blind randomised controlled trial. Participants are recruited at hospital antenatal clinics and other NHS settings throughout England and Wales or via social media advertising. Those enrolled are in antenatal care, <25 weeks’ gestation, smoke ≥5 daily cigarettes; accept referral for NHS stop smoking support and agree to set quit dates, try NRT and vape less than daily. Participants are randomised to: (1) usual care (UC) or (2) UC plus an intervention combining (1) NRT for preloading, (2) counselling to continue NRT during brief smoking lapses, and for those who cannot stop, (3) NRT to reduce smoking. The primary outcome is biochemically validated, smoking abstinence from 6 weeks after randomisation to 36 weeks gestation. Secondary outcomes include birth outcomes and cost per quality-adjusted life year. Questionnaires collect follow-up data augmented by medical record information. We anticipate quit rates of 10% and 15.9% in the control and intervention groups (OR=1.7). By recruiting 1430 participants, smoking, nicotine and pregnancy 3 should have 90% power (alpha=5%) to detect this effect. We will use the Economics of Smoking in Pregnancy model to estimate cost-effectiveness.

**Ethics and dissemination:**

Ethics approval was granted by the West Midlands—Coventry & Warwickshire Research Ethics Committee (REC reference: 21/WM/0172; Protocol number 21001; IRAS Project ID: 291236). Written informed consent will be obtained from all participants. Findings will be disseminated to the public, funders, relevant practice and policy representatives and other researchers.

**Trial registration number:**

ISRCTN84798566.

**STRENGTHS AND LIMITATIONS OF THIS STUDY**

- This randomised controlled trial is testing an intervention to enhance smoking cessation care for pregnant women, where pregnant women will be offered nicotine replacement therapy: (1) to reduce smoking before quitting (‘preloading’), (2) during brief smoking lapses after quitting and (3) to help those who cannot stop smoking, to reduce instead.
- The trial design is explanatory and pragmatic and thereby will show whether changes in smoking are due to the intervention.
- Trial design is reported according to the Standard Protocol Items: Recommendations for Interventional Trial guidelines.
- Participants are not blind to the treatments; we will aim to limit this bias by using biochemical validation of abstinence as the primary outcome.
- Obtaining data on smoking status in pregnancy is difficult; using routine data may help improve this.

## Introduction

 Smoking in pregnancy (SIP) is the biggest reversible cause of miscarriage, stillbirth, prematurity, low birth weight (LBW), perinatal, neo-natal, sudden infant death and poorer infant cognition and behavioural outcomes.^1^,^3^ Stopping SIP reduces LBW and neonatal intensive care admissions, resulting in healthier babies.^4^ SIP is a major international public health problem; in high-income countries, prevalences are between 13% and 25%,^5^,^8^ and a similar epidemic is developing in low- and middle-income countries.^9^ In England in 2017/2018, when this study was planned, 11% of women were smoking at childbirth with rates highest in economically deprived areas (Blackpool 26%).^10^ This translated to around 94 600 SIP-exposed fetuses annually in England and Wales.

Nicotine replacement therapy (NRT) is effective for smoking cessation in pregnancy (risk ratio (RR; 95% CI) for stopping with NRT compared with control 1.41 (1.03 to 1.93))^11^ and is very probably safer than smoking.^11 12^ However, NRT appears to have more impact on quitting by non-pregnant smokers (RR (95% CI) 1.55 (1.49 to 1.61)). This disparity is likely explained by pregnancy-related acceleration in nicotine metabolism, which means higher nicotine doses are needed to prevent cravings, and so help pregnant users to abstain.^13 14^ As NRT is one of the few treatments that can be used in pregnancy and is offered to pregnant women by all UK Stop Smoking Services (SSS),^15^ seeking ways to maximise NRT effectiveness is imperative.

Outside of pregnancy, NRT helps more people to stop smoking when, used as ‘preloading’, ‘smoking reduction’ or ‘lapse recovery’. ‘Preloading’ involves using NRT, such as a nicotine patch, while still smoking and preparing for a quit date (QD) in 2–4 weeks; this improves smoking abstinence at 6 months compared with usual care (UC) (RR (95% CI) 1.25 (1.08 to 1.44))^16^ and effects may persist for longer.^17 18^ ‘NRT smoking reduction’ comprises using NRT to smoke fewer cigarettes; without necessarily intending to quit, those who try this are more likely to subsequently stop smoking completely compared with those who try to reduce without NRT (RR (95% CI) for cessation 1.87 (1.43 to 2.44)).^19^ NRT is used as ‘lapse recovery’ during brief lapses to smoking or ‘slip ups’ that occur after the QD. NRT lapse recovery consists of continuing rather than stopping NRT during these slip ups. With NRT lapse recovery, quitters who lapse are more likely to return to complete abstinence. A secondary analysis from a placebo-controlled randomised controlled trial (RCT) found that quitters who used NRT rather than placebo during lapses were more likely to be abstinent 6 or 10 weeks later ((8.3% vs 0.8%; RR, 11.0, 95% CI (2.59 to 48.72) and (9.6% vs 2.6%; RR, 3.7, 95% CI (1.61 to 8.43)), respectively.^20^ Additionally, a trial testing NRT for lapse recovery found a non-significant 5% higher 4-month quit rate (51% vs 46%) in those who used NRT in brief smoking lapses.^21^

NRT used as ‘preloading’ or ‘smoking reduction’ outside of pregnancy results in lighter smoking and less exposure to tobacco smoke toxins.^22^ Studies investigating concurrent NRT use and smoking by pregnant women suggest that both uses of NRT would reduce smoking and tobacco smoke toxin exposures in pregnancy too. During the ‘titration’ phase of an RCT testing nicotine patches for quitting in pregnancy,^23^ participants used NRT patches to cut down smoking. Compared with when smoking, women who used NRT patches but still smoked, used fewer weekly cigarettes (mean difference (MD) −48, 95% CIs −52 to −38) and exhaled less carbon monoxide, CO (MD −3 ppm, 95% CI −4 to − 2ppm) but had similar cotinine concentrations (118 ng/mL vs 111 ng/mL, MD (ratio of) geometric means, 0.94, 95% CIs 0.83 to 1.07).^24^ Similarly, from a cohort of 32 women who were in the first month after quitting, 24 submitted daily reports of NRT use and smoking heaviness to a self-monitoring app. In the 17 who admitted both using NRT and smoking on at least 1 day, we found a strong, inverse relationship between smoking heaviness and NRT dose on those days^25^; for each 10 mg increase in nicotine from NRT, the daily number of cigarettes smoked fell by 0.6 (24% reduction).

### Rationale

Despite the promising findings in non-pregnant populations, we could find no trials conducted in pregnant people, which investigated the effectiveness of NRT used for preloading, lapse recovery or for smoking reduction. Hence, in smoking, nicotine and pregnancy 3 (SNAP3), we combined all three NRT uses into a single intervention and tested the effectiveness and cost-effectiveness of adding this to UC, as compared with UC alone. In this paper, we report the protocol for the SNAP3 Trial, according to the Standard Protocol Items: Recommendations for Interventional Trial guidelines.^26^

## Methods and analysis

SNAP3 is a two-arm, parallel, open-label, individually RCT recruiting from antenatal settings and via online advertising.

### Objectives

#### Primary objective

To compare the effectiveness of UC plus enhanced support for preloading and lapse recovery and NRT offered for preloading, with UC alone for promoting prolonged smoking cessation in pregnancy.

#### Secondary objectives

1. To investigate the incremental cost-effectiveness of UC plus enhanced support for preloading and lapse recovery and NRT offered for preloading, with UC alone for prolonged smoking cessation in pregnancy.
2. To compare the effects of UC plus enhanced support for preloading, lapse recovery and smoking reduction and NRT offered for preloading and smoking reduction with UC alone on:
  - Validated 7-day abstinence from smoking in late pregnancy.
  - Reported 50% reduction in smoking.
  - Exhaled CO at delivery.
  - Maternal, fetal and neonatal birth outcomes.

### Eligibility criteria

#### Inclusion criteria

Pregnant people of any age are eligible if they are <25 weeks’ gestation; have been referred for or have been receiving antenatal care; smoke ≥5 daily cigarettes; are willing to set a QD and accept referral to SSS; are willing to use NRT patch to try to stop smoking; are able to understand written and spoken English and have the capacity to give informed consent.

#### Exclusion criteria

People are not eligible if they are already in a cessation study; have absolute contraindications to NRT use such as known severe reaction/hypersensitivity to NRT, recent cardiovascular/cerebrovascular event or changes and taking theophylline/clozapine; or currently use and intend to continue to use e-cigarettes every day.

### Recruitment

Participants will be identified from National Health Service (NHS) clinical settings in England and Wales, or via online advertisement outside of the NHS settings. A list of all trial sites can be found here: https://www.isrctn.com/ISRCTNISRCTN84798566.

#### Participant identification

##### NHS settings

NHS settings can include hospital antenatal care, community midwifery care, general practice (GP) or SSS settings. NHS settings can be sites where staff identify and recruit participants or ‘patient identification centres’ where site staff identify potential participants who are then offered enrolment by the central trial team. The site principal investigator (PI) can be a clinician or a research midwife/nurse.

Potential participants can be identified from patient records and lists of women booked for antenatal care/clinics or referred for smoking cessation; staff can approach patients as they attend appointments or by telephone, letter, email or text. Posters advertising the study, with a link to the patient information sheet (PIS) and research team’s contact details, can be displayed in surgeries and clinics.

Screening questionnaires and summary leaflets can be distributed to potential participants by post or electronically to facilitate approaches described above. These can help identify pregnant people eligible for study entry. Potential participants interested in joining the study may provide their contact details either using wet-ink screening questionnaires or electronically, via a link to a secure REDCap database, hosted by the University of Nottingham.

GPs can send text messages containing similar links to potential participants identified via periodic medical record searches.

NHS settings may also use digital advertisements on their social media, electronic patient notes and websites; these include links to the study PIS and screening questionnaires.

##### Online advertisement outside of the NHS settings

Digital advertisements with an embedded link to the electronic screening questionnaire, targeted at people who are pregnant and smoke, will be published periodically on Google and social media, such as Facebook. Potential participants who complete the screening questionnaire will receive a text and/or an email with the link to the PIS and consent form. The research team will contact them over the telephone to answer any questions, discuss eligibility and to consent them to the study.

### Consent

Consent can be obtained by the PI or delegate, either face to face or over the telephone, using wet-ink or electronic consent form, as appropriate. An electronic consent form is generated by the researcher via a secure REDCap database; participants will be able to sign electronically on a device, such as a mobile phone or a computer. Verbal telephone consent via REDCap generated form can be obtained if the participant is unable to access the electronic consent form. A strict protocol will be followed for all consent procedures, and copies of consent will be shared with the participant. Subsequently, letters will be sent to all participants’ GPs to inform them of the study enrolment.

### Randomisation and blinding

Once informed consent is documented, the researcher will collect baseline data, before randomising the participant to either the intervention or control group in a 1:1 ratio, using the Nottingham Clinical Trials Unit’s web-based randomisation system. The randomisation schedule is stratified by site and level of nicotine addiction and is based on a computer-generated pseudo-random code using random permuted blocks of varying size.

Participants will be aware of their treatment allocation, but researchers who collect primary outcome data will be blinded. Staff randomising participants will not be blinded to treatment allocation, as they will need to ensure participants in each group receive relevant treatment information and referral, if required.

The trial statistician will remain blinded prior to database lock and will be unblinded prior to conducting the final analysis.

### Intervention

With no restriction on treatments used, we will offer participants in both groups the usual NHS stop smoking support available to them. The components and intensity of cessation support available vary across localities.

#### Usual care

##### Postenrolment

After randomisation, as per standard NHS practice, recruiting staff will provide ‘Very Brief Smoking Advice on Smoking for Pregnant Women**’** (VBA), as defined by the National Centre for Smoking Cessation Training (NCSCT).^27^ Staff are trained to NCSCT standards and VBA comprises:

- Exhaled air CO monitoring,
- Advice that intensive behavioural support increases chances of quitting, and
- Referral for this*,* or other available NHS support.

Participants will also be provided with a standard smoking cessation leaflet, “Smoking and your baby: advice for parents”, which comprises advice on stopping smoking and creating smoke-free environments as well as help in finding local NHS support to stop smoking.

##### Referral for NHS stop smoking support

After being enrolled and receiving VBA, participants will be referred for locally available NHS stop smoking support, which can be offered at hospitals, GP surgeries, pharmacies or from helplines, and we note the characteristics of support offered in different areas. Details for the NHS Smokefree Website, which is a further source of support and advice, will also be provided.

In most study areas, participants can access intensive behavioural support delivered by NHS stop smoking practitioners (SSPs), which follows NCSCT guidance and contains these common components^3 28^:

- Help setting a QD,
- Face-to-face or telephone/video counselling, and
- NRT starting on a QD when smoking abstinence begins.^15 29^

Letters will be sent to GPs, informing them of participants’ enrolment in the study and asking them to consider prescribing cessation-orientated NRT as per current NICE guidance if participants request this.

In usual NHS support, NRT is used for cessation only, and not for smoking reduction or preloading; quitters are advised to stop NRT if they re-start smoking.

### Intervention

The study intervention comprises three components delivered by dedicated SNAP3 SSPs, trained to the required NCSCT standard for delivering stop smoking support in the UK NHS.^30^ For NRT, Nicorette Invisipatch 15 mg/16 hours patch and Nicorette 15 mg inhalator will be used.

#### Component 1: preloading

Preloading advice will be delivered before participants try stopping smoking (before QD), and comprise:

- Up to 4 sessions of preloading counselling,^17^ tailored for pregnancy.
- For 1–4 weeks before a QD, a daily 15 mg/16 hours nicotine patch.
- A hard copy preloading leaflet with links/QR code to an identical online version; this comprises information on benefits of using NRT patches for preloading, safety of NRT, advice on how to use NRT patches for preloading and after QD, managing cravings, lapse recovery and study team contact details.
- Personalised text messages reinforcing preloading principles.

In the first counselling session, SSPs will discuss concerns about nicotine and NRT side effects and help participants set a QD for between 1 and 4 weeks’ time. While in previous studies, preloading participants were advised to wear the NRT patch while smoking as usual,^17^ in SNAP3 participants will be counselled to preload by wearing NRT patches and reducing daily smoking in the run up to their QD. Up to three more weekly counselling sessions will be offered, with the total number corresponding to time spent preloading. Each week, SSPs will send participants an average of two personalised motivational/adherence enhancing texts and one consultation reminder. In localities where further NHS stop smoking support is available, after the first preloading session, SSPs will refer participants for follow-on cessation support, aiming for continuity of treatment. Where there is no local NHS cessation-orientated support, SSPs will advise participants to contact their midwife, pharmacy or GP and inform participants about the NHS Smokefree Website.

#### Component 2: lapse recovery counselling

During the preloading period, SSPs will also advise participants to continue NRT during any brief lapses to smoking that may occur after their QDs. If lapses persist for 14 or more days or participants smoke more than 5 cigarettes for 3 or more days, participants will be advised to stop using NRT, end the quit attempt and contact their local NHS stop smoking support for help making a new quit attempt.

The leaflet, website and personalised texts described above will be used to reinforce lapse recovery counselling messages.

#### Component 3: NRT for smoking reduction

At 6 weeks postrandomisation, participants who still smoke despite being offered the first two intervention components and/or NHS support to stop smoking will be offered referral for NHS stop smoking support. Those who decline will be offered the third intervention component, NRT for smoking reduction.

The aim of this component is for participants to reduce their smoking such that they smoke fewer cigarettes than at enrolment and fewer than 10 daily cigarettes (whichever is the lowest); infants’ birth weight reductions are greatest below this smoking intensity.^31^

This intervention component comprises:

- Biweekly telephone or video counselling to use NRT for smoking reduction.
- 2 week supplies of Nicorette Invisipatch 15 mg/16 hours nicotine patches or Nicorette 15 mg NRT inhalator cartridges (up to 6 daily) until childbirth, contingent on sufficient smoking reduction.
- A hard copy reduction leaflet with links/QR code to an identical online version.
- Text messages reinforcing reduction principles.
- Use of a bespoke smartphone app, called ‘MyNRT’, for self-monitoring smoking and NRT use.

### Data collection

**Figure 1:**
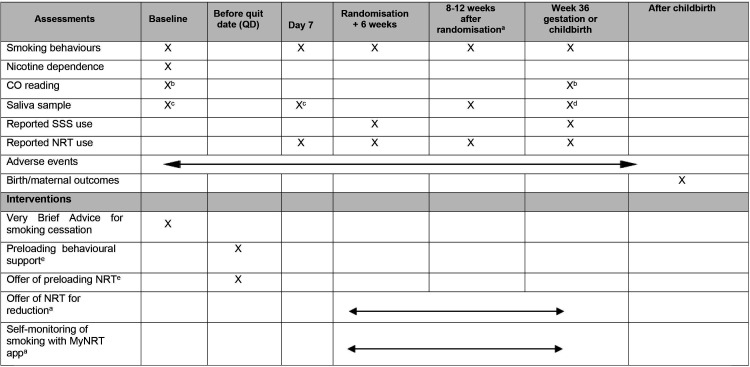
Schedule of data collection and intervention delivery time points. ^a^Participants who accepted component 3 of the intervention—Reduction. ^b^CO reading collected directly during face-to-face appointment or from a recent antenatal appointment record. ^c^Intervention group only, until approximately 30 paired samples obtained. ^d^Validation sample, only collected from participants who reported not smoking in the last 7 days. ^e^Intervention group only. CO, carbon monoxide; NRT, nicotine replacement therapy; SSS, Stop Smoking Services.

**Figure 2:**
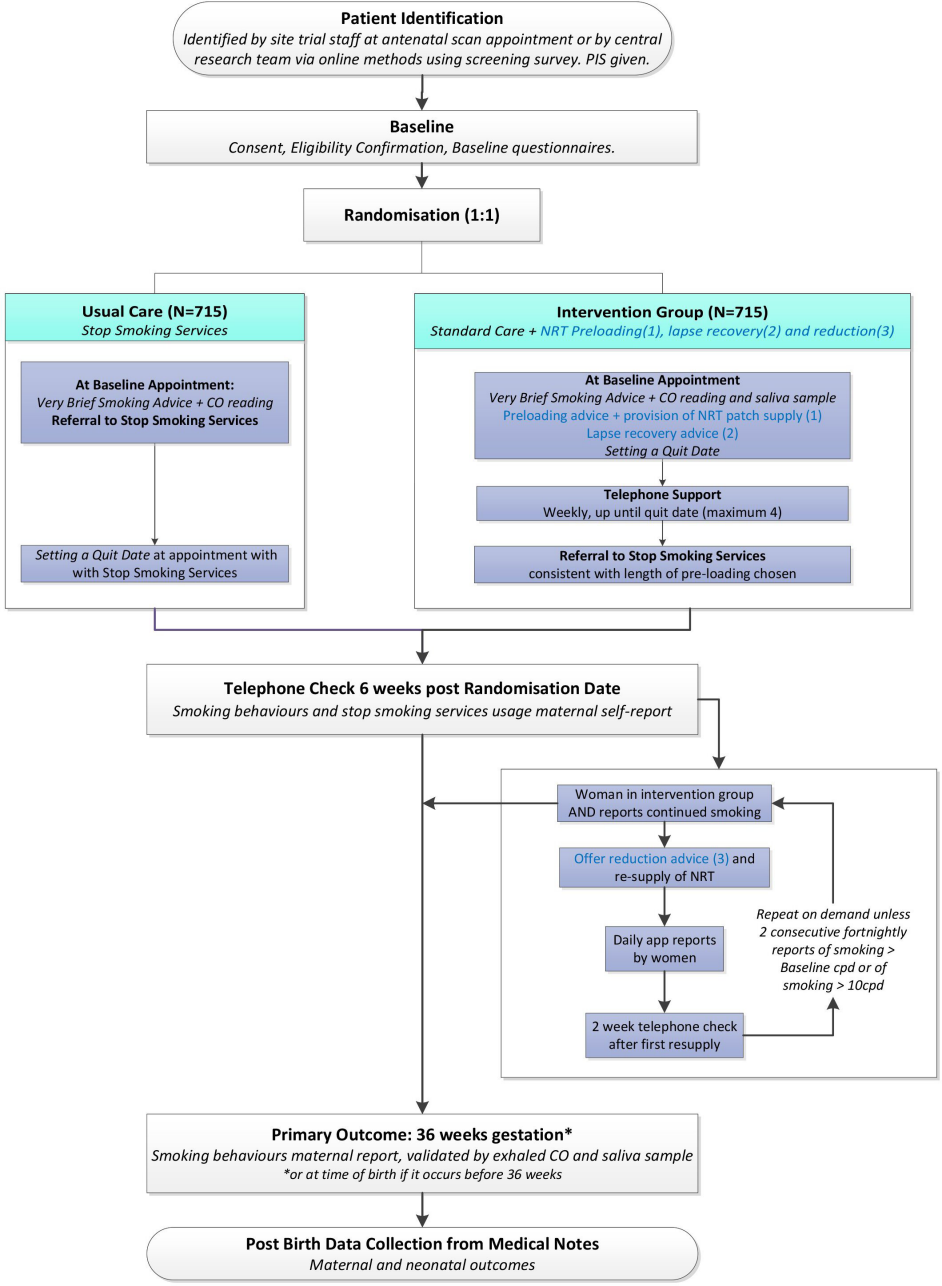
Study flow diagram. CO, carbon monoxide; cpd, cigarettes per day; NRT, nicotine replacement therapy; PIS, patient information sheet;

Figure 1 shows all participant data collection at time points outlined below and indicates how intervention delivery fits with this. Figure 2 is a study flow diagram.

#### Baseline

Researchers will collect the following baseline data: socio-demographics; smoking beliefs and behaviours; gestation; nicotine dependence^32^; urges to smoke; number of births beyond 24 weeks and significant others’ smoking status. Those recruited in person will be asked to provide an expired air CO reading, and for those recruited remotely we will seek a recent CO reading from antenatal appointment records. For those in the intervention group, we will ask them to provide a saliva sample. For participants recruited remotely, we will post out self-collection saliva sample kits, and samples will be posted directly to the study laboratory for cotinine measurement. (Please note, identical sample collection procedures will be used at all time points).

#### Follow-up

Where possible, data will be first sought via online questionnaires, and if unsuccessful, by telephone, email, text or postal questionnaire. Data collection will occur:

- 7 days after preloading starts (intervention group only).
- 6 weeks after randomisation.
- 8–12 weeks after randomisation (for those who accept the NRT for smoking reduction intervention only).
- At 36 weeks gestation (or childbirth, if earlier).
- After childbirth.

#### Day 7 of preloading intervention

We will ask intervention group participants how many daily cigarettes they smoke, about use of preloading NRT and for a saliva sample; baseline and day 7 saliva sample collection will end when we have around 30 paired samples.

#### Randomisation plus 6 weeks

We will ask about smoking status, quitting behaviour, use of preloading NRT, whether a QD was set and use of non-study stop smoking support. For those who report a quit attempt, we will ask if they used NRT for quitting, and if any smoking lapse(s) occurred, whether NRT was continued or stopped. To prevent researchers’ becoming aware of participants’ treatment allocations, which could bias questioning about smoking, questions about smoking will be asked at the very outset, before questions about treatments.

#### Offering NRT for smoking reduction

We will aim to offer the third intervention component to all intervention group participants who are smoking and not interested in trying to quit. Hence, if follow-up is by telephone, we will offer this during the call; alternatively, we will contact those participants who report smoking in their responses to online questionnaires. Those who accept NRT for help with smoking reduction, and for whom there is no baseline saliva sample, will be asked to provide one before they start using NRT for reduction.

#### 8–12 weeks postrandomisation

We will ask those who accepted NRT for smoking reduction how many daily cigarettes they smoke, about use of preloading NRT, and to provide a saliva sample and an exhaled air CO sample.

#### 36 weeks gestation (or childbirth, if earlier)

We will ask about smoking behaviours and quit attempts, NRT and e-cigarette use, use of stop smoking support and about relevant cost data. We will attempt to collect saliva samples from all who report abstinence from smoking for at least 7 days. Samples will be requested postally (see above) or at hospital attendance and before collection we will ask, by questionnaire or verbally, about smoking and recent use of NRT or e-cigarettes.

#### After childbirth

Most data collection at this point will involve hospital staff extracting data from participants’ medical records.

##### Birth and maternal outcomes

We will seek maternal and fetal pregnancy outcome data, and information on infant gender, birth weight, gestation at delivery, mode of delivery, admission to neonatal intensive care, congenital anomalies or neonatal death. *Smoking outcomes*: We will seek smoking status and CO readings recorded in electronic medical records at 36 weeks, the time of delivery and from any clinic appointments occurring close to study enrolment.

Where birth and late pregnancy smoking data are still missing after this final attempt at data collection, we will seek them directly from participants via text message or telephone.

##### Incentives for follow-up completion

Participants will be offered £5 high street shopping vouchers for returning completed follow-up questionnaires, and a £20 voucher for providing validation saliva samples at 36 weeks.

### Qualitative substudy to optimise recruitment

Early in the trial, we will interview up to 28 women who are offered trial enrolment. We will aim to include approximately 10 participants from each arm and up to eight who decline participation. We will design and pilot interview guides for participants and non-participants and interview them, asking about acceptability of being offered participation, perceived barriers against and facilitators for joining the study, and whether approaches to potential participants could be improved. Participants will also be asked about acceptability of and concerns about intervention components. Interviews will be recorded and transcribed, and interviewees will be offered a £20 shopping voucher as compensation for their time. There will be a pragmatic, thematic analysis, with findings hopefully informing study modifications that will enhance recruitment.

### Data management and archiving

Participants will be assigned unique study identification numbers used to identify their data and biological samples. Identifiable data, such as name/date of birth/contact details, will be stored in a password-protected database accessible only by the research team. If a paper case report form (CRF) is used, data will be entered by researchers onto the database and the paper CRF will be stored in a secure location in the investigator site file. Information submitted by participants via the MyNRT app will be stored as pseudonymised data on Amazon cloud.

Data Management is led by Nottingham Clinical Trials Unit, with the support of the Trial Manager in the University of Nottingham as detailed in the Data Management Plan.

The Trial Management Group will be responsible for the day-to-day running of the trial, meeting on a regular basis and will be supported by, and report to, the Trial Steering Committee (TSC) and the Data Management Committee (DMC).

Saliva samples will be analysed at the end of the trial by ACM Biomedical Services, an external laboratory to quantify salivary cotinine concentrations and the presence or absence of anabasine using a quantitative enzyme immunoassay technique. After analysis, samples will be destroyed in accordance with the Human Tissue Act 2004.

In compliance with the The Global Health Network’s International Council for Harmonisation (ICH) Good Clinical Practice (GCP) guidelines/regulations and in accordance with the University of Nottingham Research Code of Conduct and Research Ethics, the chief or local principal investigator will maintain all records and documents regarding the conduct of the study. These will be retained for at least 7 years following the end of study data lock or for longer if required. If the responsible investigator is no longer able to maintain the study records, a second person will be nominated to take over this responsibility.

The trial master file and trial documents held by the chief investigator on behalf of the sponsor shall be finally archived at secure archive facilities at the University of Nottingham. This archive shall include all trial databases and associated meta-data encryption codes.

## Outcomes

### Primary outcome

Biochemically validated, prolonged abstinence from smoking reported between 6 weeks after randomisation and 36 weeks gestation or childbirth, whichever is earlier.

Smoking lapses are permitted during this period, provided no more than five cigarettes in total are smoked and a 7-day period of total abstinence is reported immediately before primary outcome follow-up.

### Secondary outcomes

#### Smoking-related

- Reported and validated 7-day smoking abstinence at 36 weeks gestation
- Reported ≥50% reduction in daily cigarettes at 36 weeks gestation
- Exhaled CO concentration at delivery
- Brief smoking lapse(s) reported at 6 weeks postrandomisation

#### Pregnancy and safety-related

- Miscarriage
- Stillbirth
- LBW for gestational age
- Unadjusted birth weight and birth weight z score
- Premature birth
- Gestation at birth
- Congenital abnormality
- Mode of delivery
- Admission to special care
- Neonatal death

#### Exploratory outcomes

- Exhaled CO and cigarettes smoked per day when using NRT for ‘preloading’ or to cut down smoking
- Saliva cotinine concentration when using NRT for ‘preloading’ or to cut down smoking
- Use of stop smoking support

### Sample size and justification

To detect a difference in smoking cessation in late pregnancy between control and intervention group proportions of 0.1 and 0.159, respectively, (OR of 1.70) with 90% power and 5% two-sided alpha requires 715 participants per arm (total n=1430). There is no adjustment for missing data; it will be assumed those lost to follow-up are smoking. Based on a recent SiP trial,^33^ assuming a quit rate in the UC group of 10% and anticipating an OR of 1.70 for the effects of NRT (1) as preloading and (2) continued in smoking lapses to facilitate return to abstinence. An OR of 1.7 is considered clinically important and plausible, based on the anticipated effect of *preloading*^17 34^ and *lapse prevention*^21 35 36^ intervention components. The third intervention component (*NRT for smoking reduction*) will not contribute to the primary outcome as this is offered to participants who have returned to smoking.

#### Exploratory outcomes

We intend to use paired saliva samples to estimate participants’ cotinine concentrations when (1) smoking at baseline and using NRT for (2) preloading and (3) smoking reduction.

## Analyses

Descriptive statistics of demographic and clinical measures will be used to assess balance between the randomised arms at baseline, but no formal statistical comparisons will be made. Continuous variables will be summarised in terms of the mean, SD, median, lower and upper quartiles, minimum, maximum and number of observations. Categorical variables will be summarised in terms of frequency counts and percentages.

The primary between-group analysis will use the intention-to-treat population, with those lost to follow-up considered to be smoking. The primary outcome will be analysed using a mixed-effect logistic regression model adjusted for randomisation stratification variables with a random effect for site. The estimate between-group effect will be presented using both absolute and relative measures of effect, with associated 95% CIs.

Maternal secondary outcomes will be analysed similarly, using appropriate regression models depending on the type of outcome variable and adjusting for randomisation stratification variables. Infant secondary outcomes will be analysed using appropriate regression models depending on the type of outcome variable, adjusting for randomisation stratification variables and accounting for correlation between outcomes for infants from multiple pregnancies.

Primary analysis will be repeated additionally, adjusting for any variables with marked imbalance at baseline.

Using appropriate interaction terms in the primary regression analyses for prolonged smoking cessation, it will be investigated whether treatment effectiveness differs according to the baseline level of nicotine dependence. Since the trial is powered to detect overall differences between the groups rather than interactions of this kind, this subgroup analysis will be regarded as exploratory.

Further exploratory analyses will be carried out for women in the intervention arm who are offered NRT for (1) preloading and (2) smoking reduction; these will describe saliva cotinine and exhaled CO concentrations when smoking with those when (1) preloading and (2) using NRT to reduce smoking. Previous studies suggested that it would be reasonable to collect 30 paired samples for this analysis.^37^ Another exploratory analysis will describe and compare the characteristics and outcomes of women who accept and do not accept NRT for smoking reduction and cessation induction. This will be the first study of pregnant women to be offered NRT for smoking reduction as part of a trial.

Full details of analyses will be provided in a statistical analysis plan (SAP), which will be finalised and approved prior to the database lock and before the trial statisticians are unblinded. This will then be added to the ISRCTN registry.

### Assessment of efficacy

For the primary outcome, number and percentage of participants achieving biochemically validated, prolonged abstinence from smoking reported between a QD and 36 weeks gestation or childbirth, whichever is earlier, will be reported for each treatment group. For this outcome, smoking lapses are permitted during this period, provided no more than five cigarettes in total are smoked^38^ and a 7-day period of total abstinence is reported immediately before primary outcome follow-up. Reported abstinence will be validated by exhaled CO, saliva cotinine or saliva anabasine, with the actual measure used depending on abstinent participants’ reported use of NRT and/or e-cigarettes.

### Procedures for missing, unused and spurious data

For the primary analysis of the primary outcome and for other smoking-related secondary outcomes, participants with missing data will be considered to still be smoking; this is consistent with Russell Criteria, which defines standard smoking cessation study outcomes^38^ and has been proven a rational assumption in similar contexts.^39^ However, sensitivity analyses (described in detail in the SAP) will be conducted, which make different assumptions to investigate the potential impact of missing smoking abstinence data.

Analyses of other secondary outcomes will use available data, that is, with no imputation for participants with missing data.

### Safety

The study tests a behavioural intervention aimed at improving effectiveness of NRT use for smoking cessation in pregnancy. NRT is a standard NHS treatment and therefore we do not anticipate any harm being caused. We do, however, monitor any adverse events occurring during study participation. Data for pregnancy-related secondary outcomes listed above, and adverse events, separated by trial arm, will be reported to the DMC for their consideration. This will allow us to detect unexpected impact on the pregnancy and fetus to be detected and responded to while the trial is still ongoing. Although such impact is unlikely, it is important to stay vigilant to the possibility.

### Economic analysis

A cost-utility analysis will be performed from an NHS and Personal Social Services perspective^40^ using a previously published and peer-reviewed decision analytic model (DAM).^41 42^ Intervention costs, including staff time for training and intervention delivery, as well as costs associated with materials and prescriptions, will be collected alongside other trial data, and estimated at most recent price year to give a per participant cost for intervention and control arms. The DAM will be programmed with the following trial results; number of women included in intention-to-treat analysis, year of birth, average age of mother, cost per participant for intervention and control arms, and biochemically validated prolonged abstinence rates for intervention and control arms. The DAM will extrapolate trial results up to age 100 years for the mother and up to age 100 years for her offspring, including smoking behaviour, healthcare costs, life years gained and quality-adjusted life year (QALY). Primary measure of cost-effectiveness will be the incremental cost-effectiveness ratio (ICER) per QALY gained for combined mother and offspring lifetimes. Secondary measures of cost-effectiveness will be ICERs per additional quitter, per life year gained and per QALY gained, for mother and offspring outcomes at end of pregnancy and lifetime, both separately and combined. To control for uncertainty, the DAM will perform a probabilistic sensitivity analysis using 10 000 Monte Carlo simulations, which will estimate 95% CIs for ICERs, cost-effectiveness scatterplots and cost-effectiveness acceptability curves. To demonstrate return on investment, the DAM will estimate cost-offset ratios defined as the incremental healthcare savings (difference in healthcare costs between comparator and intervention groups plus incremental intervention costs) divided by incremental intervention costs, to estimate a return for every one Great British Pound (GBP) spent on the intervention.

### Patient and public involvement

A patient and public involvement (PPI) group has been involved in the trial design and throughout the study duration. The group consists of women who had experience of smoking during pregnancy. In the early stages of the intervention design, we interviewed five PPI group women who had experience of pregnancy and smoking. We discussed the proposed components and made changes to the intervention based on the comments and ideas brought by the PPI group. All interviewed thought NRT use for preloading and lapse recovery can be beneficial for pregnant people who wish to try and stop smoking. However, there was some concern about allowing women to use NRT throughout pregnancy without any focus on abstinence. In response to this point, the third intervention component was amended such that researchers will monitor participants’ heaviness of smoking and will stop NRT provisions if women admit smoking more heavily than at baseline or >10 daily cigarettes. This change was made to ensure a focus on reducing smoking when using NRT, rather than on ‘smoking as normal’.

During study set-up, representatives of the PPI group are being consulted on all aspects of the study, such as acceptability and accessibility of participant-facing documents, recruitment and retention methods, methods of data collection and the dissemination of findings. To ensure PPI involvement in the project’s strategic direction, two PPI representatives independent from the study team are invited to attend each TSC meeting.

### Ethics and dissemination

Ethics approval was granted by the West Midlands—Coventry & Warwickshire Research Ethics Committee (REC reference: 21/WM/0172; Protocol number 21011; IRAS Project ID: 291236). Approval for any protocol modifications will be sought from the REC/HRA and communicated to the funder and trial registry. Written informed consent will be obtained from all participants. Findings will be disseminated to the public, funders, relevant practice and policy representatives and other researchers. A data-sharing agreement has been written. Once the trial has finished and the main trial paper has been published, a de-identified data set will be available on reasonable request from the Nottingham Clinical Trials Unit.

### Trial and recruitment status

Recruitment began in May 2022 and is planned to continue until June 2026, and after all follow-up will be concluded, trial results are currently expected in mid-2027.

The study start was delayed by 1 year due to COVID-19, and implementation in the post-COVID NHS has been challenging; the timeline above includes a study extension. By 30 September 2025, 930 participants had been enrolled.

An ‘in trial’ pilot phase to demonstrate to funders that the full trial is deliverable was planned for the first 9 months of recruitment. Due to changes to the delivery of the NHS stop smoking care to pregnant women and COVID-19, at the end of the 9-month period, the trial did not reach the anticipated recruitment target of 385 participants. The NIHR Health Technology Assessment (HTA; the funder) reviewed the trial progress at that point, and the trial advanced beyond the pilot phase, with a further set of recommendations set up by the funder. Progression against the new targets was reviewed again 9 months later, and the trial team were invited to request a funded extension. The extension was granted in September 2023, for an additional 14 months of recruitment period, until December 2025. In August 2025, a further non-cost extension has been approved for an additional 6 months of recruitment. The recruitment end date is planned for 30 June 2026. The follow-up will continue for a further 10 months, with the participant’s last visit due in April 2027. There has been a marked improvement in site set-up and participant recruitment from 2023. Due to the COVID-19 pandemic, recruitment via the traditional routes became problematic; in response to those issues, early in the study we have developed alternative routes of recruitment online. We have successfully established and continue to use a pathway of recruitment via social media advertisement. We initially planned to open 26 recruiting sites, but after the review this was increased to 30 sites.

Protocol version: 5.0, 19 October 2023. For protocol changes see supplementary materials.

## Supplementary material

10.1136/bmjopen-2025-109568 online supplemental file 1
