# Supplementary material for: Smoking, nicotine and pregnancy 3 (SNAP3) trial: protocol for a randomised controlled trial of enhanced support and nicotine replacement therapy (NRT) offered for preloading, lapse recovery and smoking reduction in pregnancy
Source: BMJ Open. 2025 Nov 13;15(11):e109568. doi: 10.1136/bmjopen-2025-109568 (PMC12625952; doi:10.1136/bmjopen-2025-109568)
Supplement: online supplemental file 1 [file bmjopen-15-11-s001.docx]

SNAP3 Trial - List of changes made to previous protocol versions

| **Amendment number** | **Date of Amendment** | **Summary and version number** |
| --- | --- | --- |
| NSA01 | 07 Dec 2021 | **Protocol v1.1:**  Version 1.1 10Nov2021  **(1) Changed Trial Statistician.**  **(2) Removed "confidential" from footer.**  **(3) Changed the start date to 1 Oct 2020**  **(4) Removed the reference to months 7-15 when describing the pilot period.**  **(5) Added the NIHR disclaimer.**  **(6) Corrected eligibility criteria should be "<25 weeks gestation";**  **(7) ISRCTN number added, (8) Change to Figure 1 now reads "6 weeks after randomisation" instead of "after quit date",**  **(9) In Table 2, changed the description of reason for using NicUse App**  **(10) Few minor changes to the text.** |
| SA01 | 16 Feb 2022 | **Protocol v2.0:**  Version 2.0 07Feb2022  **Staff contact details**  **Secondary objectives –** removed CO validation of 50% reduction in late pregnancy  **Exploratory outcomes:**  Added “cigarettes per day (CPD) when using NRT for preloading or to cut-down smoking”  Removed “reported use of NRT before the quit date and in brief smoking lapses” (now process measure)  **Inclusion criteria:** changed from “scan confirmed pregnancy” to “have been referred for or have received or attended an appointment as part of standard antenatal care”.  **Exclusion criteria:** removed chemical dependence and alcohol addiction  **Details of product –** changed the number of NRT cartridges from 12 to 6.  **Progression criteria**: have now been removed and replaced with statement clearly stating there is an in-trial pilot, which will last 9 months from recruitment start, and will be reviewed by the funder and TSC at the end of the pilot.  **Recruitment and participant identification**: added Stop Smoking Services as PICs.  **Sample collection:** changes to collection of saliva sample at baseline.  **Sample analysis**: preloading/reduction samples and matching baseline samples to be analysed when required to report to the DMC for safety purposes.  **NicUse App** – remove the app name – this will have to be changed to avoid confusion with the old app version |
| NSA06 | 07/07/2022 | **Protocol v2.1:**  Version 2.1 09Jun2022  Contraindications to NRT updated: known severe reaction/hypersensitivity to NRT, recent cardiovascular/cerebrovascular event or changes and taking Theophylline / Clozapine  Typo corrected on page 29 of Protocol - 6 instead of 12. |
| SA03 | 15/12/2022 | **Protocol v3.1:**  Version 3.1 20Dec2022  1) removal of secondary outcome “reported 7 day smoking abstinence at 6 weeks after randomisation”  2) addition of general practices to act as PICs/recruiting sites using EMR searches followed by text message ‘mailouts’  3) participants identified via PICs can be recruited by site trial staff if booked to deliver at the SNAP 3 recruiting site.  4) clarification that Reduction phase offer can occur ‘At Follow up 1 *or shortly thereafter’*  5) change of statistician |
| SA04 | 07/06/2023 | **Protocol v4.0:**  1) Version 4.0 25May2023  2) Participant identification, p21: we added a paragraph to state that a Summary PIS may be  used as a cover page for the full PIS or a stand alone document, to provide potentially eligible  women with key information about the trial. Summary PIS will contain a link to the full PIS and a  screening survey.  3) Study data collection/After Child birth, p36 – We added a paragraph to describe a method of  data collection via text message, email or over the telephone – we may directly ask the  participant to answer a few questions about their birth outcomes. This will be used when  collection of birth outcomes via the CRN is not possible (e.g. online recruit or site recruit who  moved out of area). We also added a paragraph under Follow up 1, p36, that saliva and /or  CO samples may be collected for those who decide to take part in the 3rd component of the  intervention (reduction) - this is to allow us to obtain a "baseline" for comparison to "reduction"  sample obtained between weeks 8-12 for those, who did not provide a saliva sample at the  time of enrolment. |
| SA05 | 04/12/2023 | **Protocol v5.0:**  - Version 5.0 19Oct2023  - The following four events have been removed from the list of AEs that require reporting (p 41) and added to the list of events that do not require reporting as AEs (p42):  • pre-term delivery before 32 weeks,  • low birth weight (< 2,500g),  • instrumental delivery,  • caesarean section  These events should not be recorded as AEs, because they can occur in any pregnancy, and also due to the fact that they are already reported as secondary outcomes and are already reported on the CRF/eCRF as such. The trial team attempts to collect secondary outcome data from all participants, it is therefore likely that these will be more comprehensively reported as outcomes than as AEs.  If these events have already been reported as AEs according to previous protocol versions, these will be disregarded in future analysis.  In addition –a change was made to Table 2 to remove Saliva sample at baseline – this was an omission from previous amendment where we discontinued saliva sample collection at baseline. |
