# Supplementary material for: Smoking, nicotine and pregnancy 3 (SNAP3) trial: protocol for a randomised controlled trial of enhanced support and nicotine replacement therapy (NRT) offered for preloading, lapse recovery and smoking reduction in pregnancy
Source: BMJ Open. 2025 Nov 13;15(11):e109568. doi: 10.1136/bmjopen-2025-109568 (PMC12625952; doi:10.1136/bmjopen-2025-109568)
Supplement: Supplementary file 2 [file bmjopen-2025-109568.reviewer_comments.pdf]

PEER REVIEW HISTORY

BMJ Open publishes all reviews undertaken for accepted manuscripts. Reviewers are asked to complete a checklist review form and are provided with free text boxes to elaborate on their assessment. These free text comments are reproduced below.

ARTICLE DETAILS

Title (Provisional)

Smoking, nicotine and pregnancy 3 (SNAP3) trial: protocol for a randomised controlled trial of enhanced support and nicotine replacement therapy (NRT) offered for preloading, lapse recovery and smoking reduction in pregnancy

Authors

Campbell, Katarzyna A; Clark, Miranda M; Montgomery, Alan A; Partlett, Christopher; Dickinson, Anne; Bradshaw, Lucy; Jones, Matthew; Huang, Yue; Aveyard, Paul; Jiang, Yimin; Holmes, Christopher M; Coleman, Tim

---

This article was not externally reviewed at BMJ Open. Protocols that have been independently assessed prior to submission to BMJ Open are usually fast-tracked to publication on the grounds that further substantial changes will not be possible. This independent assessment will usually be external, independent review for both a substantial grant award from a non-commercial or government funder and ethics approval.
